# Liquid Metal-Based Electronics for On-Skin Healthcare

**DOI:** 10.3390/bios13010084

**Published:** 2023-01-03

**Authors:** Jinwei Cao, Xin Li, Yiwei Liu, Guang Zhu, Run-Wei Li

**Affiliations:** 1 CAS Key Laboratory of Magnetic Materials and Devices, Ningbo Institute of Materials Technology and Engineering, Chinese Academy of Sciences, Ningbo 315201, China; 2 Zhejiang Province Key Laboratory of Magnetic Materials and Application Technology, Ningbo Institute of Materials Technology and Engineering, Chinese Academy of Sciences, Ningbo 315201, China; 3 Department of Mechanical, Materials and Manufacturing Engineering, University of Nottingham Ningbo China, Ningbo 315100, China; 4 School of Integrated Circuits and Beijing National Research Centre for Information Science and Technology (BNRist), Tsinghua University, Beijing 100084, China

**Keywords:** liquid metal, epidermal healthcare, stretchable electronics, electrodes, sensors

## Abstract

Wearable devices are receiving growing interest in modern technologies for realizing multiple on-skin purposes, including flexible display, flexible e-textiles, and, most importantly, flexible epidermal healthcare. A ‘*BEER*’ requirement, i.e., biocompatibility, electrical elasticity, and robustness, is first proposed here for all the on-skin healthcare electronics for epidermal applications. This requirement would guide the designing of the next-generation on-skin healthcare electronics. For conventional stretchable electronics, the rigid conductive materials, e.g., gold nanoparticles and silver nanofibers, would suffer from an easy-to-fail interface with elastic substrates due to a Young’s modulus mismatch. Liquid metal (LM) with high conductivity and stretchability has emerged as a promising solution for robust stretchable epidermal electronics. In addition, the fundamental physical, chemical, and biocompatible properties of LM are illustrated. Furthermore, the fabrication strategies of LM are outlined for pure LM, LM composites, and LM circuits based on the surface tension control. Five dominant epidermal healthcare applications of LM are illustrated, including electrodes, interconnectors, mechanical sensors, thermal management, and biomedical and sustainable applications. Finally, the key challenges and perspectives of LM are identified for the future research vision.

## 1. Introduction

Wearable devices are of paramount importance for human life in such an era of everything being interconnected, smart, and intelligent [1,2,3,4]. In addition, they are usually employed on human epidermal skin to realize various applications such as flexible displays [5], flexible e-textiles [6,7], e-skin [8], optoelectronic skin [9], and, most importantly, human healthcare [10]. Especially in the post-pandemic era (COVID-19), more researchers are dedicated to designing distinctive devices for real-time on-body signal monitoring, including electrocardiography (ECG) [11], electromyography (EMG) [12], blood pressure [13], respiration [14], temperature [15], etc. To realize these functions, flexible conductors that possess conformability, conductivity, stretchability, and durability upon the dynamic deformations of human skin are highly required [16]. These conductors enable the perception, collection, or even procession of epidermal signals by the differentiation of electrical potential, the transmission of electrons, and the identification of mechanical deformations or chemical micro-environments as various sensors [17].

**Figure 1:**
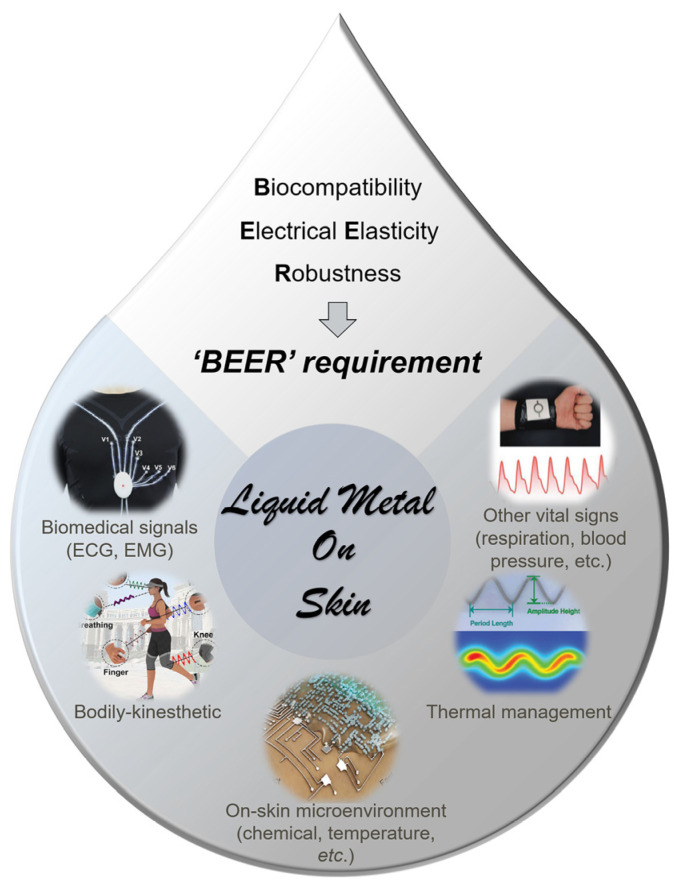
The ‘*BEER*’ requirement for flexible electronics for on-skin healthcare and the extensive applications of LM-based electronics in on-skin biomedical signals collection, on-skin microenvironment monitoring, bodily-kinesthetic recognition, thermal management, and other vital signs.

For conductive materials on the epidermal system (Figure 1), three basic requirements need to be met in fabrication and designing processes, named the ‘*BEER*’ requirement. 1. Biocompatibility: Biological compatibility is most significant for on-skin applications, especially for in vivo and in vitro biotoxicity. The conductive materials should be nontoxic to ensure the safety of the epidermal system. In addition, air-permeability and thermal comfort are important to prevent skin lesions and inflammation while ensuring long-term comfortable wearing. 2. Electrical elasticity: The conductive materials should be designed with satisfying strain tolerance to maintain their functions in on-skin scenarios. In more detail, the conductors should show stable conductivity under stretching and recovering. 3. Robustness: The epidermal system provides a dynamic deformation scenario for on-skin conductive materials. Under long-term dynamic stretching, bending, and twisting, the conductors or electrodes are supposed to maintain their conductivity for long-term use, and the biosensors should present robust sensitivity.

Through the ‘*BEER*’ requirement, a series of conductive materials and devices are being proposed and developed to constitute a smart wearable healthcare monitoring system [18]. As opposed to carbon-based conductive materials [19,20], metal-based conductive materials have sprung up with the anticipation of their high conductivity [17]. Rigid-metal conductive materials with various morphologies are emerging as processable conductive components, including nanoparticles (e.g., Au nanoparticles) [21], fibers (e.g., Ag nanowires) [22], plates (e.g., Ag flakes) [23], and their combinations (e.g., Ag nanoparticles and flakes) [24]. However, the poor toughness of rigid conductive materials would cause a dramatic drop in conductivity due to the fracture or separation of them during elongation. The reason lies in the Young’s modulus mismatch between rigid conductive fillers and a soft matrix that forms an unstable and easy-to-fail interface.

**Figure 2:**
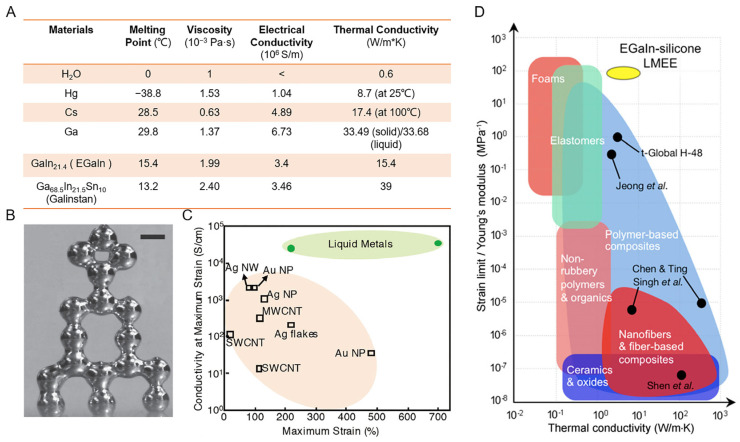
The fundamental physical properties of LMs. (**A**) Physical properties of water and typical metals. Data from Tang et al. [25], Chen et al. [26], Zhu et al. [27] and Chen et al. [28]. (**B**) A freestanding 3D tower of liquid metal droplets [29]. Copyright: (2013) WILEY-VCH. (**C**) Comparison of the maximum strain conductivities of different flexible conductors [36]. Copyright: (2018) WILEY-VCH. (**D**) Summary of the thermal conductivity for various materials as a function of the strain limit to Young’s modulus ratio [37]. Copyright: (2017) National Academy of Sciences.

Different from rigid-metal conductive materials, liquid metal (LM) with its high fluidity at room temperature is a possible solution and has emerged as a promising conductive material, e.g., mercury (Hg), francium (Fr), cesium (Cs), gallium, rubidium (Rb), etc. (Figure 2A) [25,26,27,28]. Generally, Hg is the most common but toxic, and Fr, Cs, and Rb are rare and violently reactive or radioactive, which makes them inappropriate for on-skin conductive materials. Ga-based liquid metal shows high processibility and possesses no vapor pressure, negligible solubility in water, and nontoxicity [29,30]. In addition, Ga-based LM presents excellent conductive properties of (3.4–6.7) × 10^6^ S/m under stretching [31]. These properties highlight Ga-based liquid metal as a significant intrinsically stretchable conductive material candidates that fully meets the ‘*BEER*’ requirement for epidermal electronics.

Many efforts have been devoted to design novel LM-based epidermal electronics that can be conformably attached onto skin for ECG, EMG, or EEG collection (Figure 1). Some LM-based sensors are fabricated by mimicking the function of various sensory receptors. As a result, pressure, strain, pain sensors, and other functional circuits for temperature and perspiration monitoring to serve as artificial skin have been designed [32,33,34]. In addition, blood pressure and blood pulsation in subcutaneous tissue could also be identified with a pressure sensor to monitor subtle physiological changes [35]. Prior to the applications of LMs, the representative properties of LMs will be thoroughly discussed in terms of their physical properties (conductivity, surface tension, thermal conductivity, etc.), chemical properties (oxidation, surface modification, reaction with metals, catalytic medium, etc.), and biocompatibility. In the meanwhile, various technologies for LM modification, LM patterning, circuit fabrication, and device assembly are also introduced. The challenges and opportunities of LM-based conductive materials and devices are discussed with proposed solutions and future directions. 

## 2. Properties of Liquid Metals

LMs exhibit a variety of distinct physical, chemical, and biological properties, which offer a unique application advantage in epidermal healthcare monitoring [26]. In this section, the fundamental properties of LM, including physical and chemical properties, which facilitate on-skin applications will be comprehensively introduced. The fundamental physical characteristics of liquid metal mainly consist of the melting point, electrical conductivity, fluidity, plasticity, viscosity, wettability, etc. On the other hand, the chemical properties that are related to the fabrication of LM-based circuits will be discussed, including oxidation, alloying, chemical reduction, and electrochemical reduction. More importantly, the biocompatibility of LM for in vivo and in vitro uses will be discussed to prove the feasibility of LM-based electronics for long-term epidermal applications.

### 2.1. Physical Properties

The main physical properties of water and various common LMs are presented in Figure 2A. Gallium (Ga), with its weak Ga-Ga bonds and large interatomic distance, is hence susceptible to having its crystal structure broken by heat, exhibiting a melting point of 29.8 °C. However, Ga’s boiling point is approximately 2400 °C because its p-shell contains reactive and unpaired valence electrons [38]. As a result, Ga is stable and reasonably safe to use due to its high boiling point and low melting point. Gallium can be alloyed with other metals such as Indium (In), Tin (Sn), and Cadmium (Cd). Then, the melting point of the resulting alloys can be influenced by different components and proportions. Interestingly, gallium-based binary and ternary alloys may have melting points that are even lower than those of the element itself [26]. For example, Galinstan, which is composed of 68.5% Ga, 21.5% In, and 10% Sn, melts at a low temperature of 13.2 °C and the eutectic gallium–indium alloy (EGaIn), which typically has a composition of 78.6% Ga and 21.4% In, melts at 15.4 °C.

For stretchable electronics applications, the wettability of LM is the main factor for processing possibility and feasibility on stretchable substrates. The wettability is dominantly dependent upon the viscosity and surface tension. The excellent fluidity of LM comes from its low viscosity property (1.37 × 10^−3^ Pa·S for EGaIn), which is merely twice larger than that of water [27]. Due to the high surface tension of LM (620 mN/m), LM droplets, unfortunately, have a high contact angle with a substrate and tend to be in a spherical state on the platform. Collin Ladd and colleagues demonstrate that it is possible to direct-write a low-viscosity liquid metal at room temperature into a stable free-standing 3D array of droplets by utilizing the features of low viscosity and substantial surface tension (Figure 2B) [29]. The microstructure of LM was stabilized by a thin passivating oxide coating that formed on its surface immediately.

LM shows similar electrical conductivity performance to other pure rigid metals. The electrical conductivity, the ability to conduct electric current, of LM is 3–6 × 10^6^ S/m, which is much larger than non-metallic conductive materials such as carbon-based materials, conductive paste, and conductive grease [17]. The highly conductive LM guarantees low and steady electrical resistance when working as an interconnector and circuit. Liquid metal can be injected or blended into hollow fibers and prepolymers to enable the mass production of conductors with robust stretchability and negligible stress–strain cyclic hysteresis [39,40]. The conductivity at the maximum strain of various stretchable conductors is compared in Figure 2C, where LM is located in the upper-right area that means a combination of excellent conductivity and stretchability [36].

Thermal conductivity is another important physical property of LMs. The thermal conductivity of pure Ga is 33.68 W/m·K in liquid form and 33.49 W/m·K in solid form, which is 56 times higher than that of water (0.6 W/m·K) [37]. Due to the decreased phonon transport dynamics, the elastomer’s thermal conductivity often decreases as it softens. As a result, there is a trade-off between elastic modulus and thermal conductivity. This dilemma can be solved by using LMs for thermal transport thanks to their high thermal conductivity (26.4 W/m·K at 30 °C) and liquidity. Based on this superiority, a series of thermal conductive composites were developed via elastomers mixed with LMs [41]. The resulting composites greatly outperformed any other soft materials with an amazing combination of low stiffness (100 kPa), high strain limit (>600%), and metal-like thermal conductivity (up to 9.8 W/m K) (Figure 2D). Moreover, the thermal conductivity of LMs or low melting points of alloys have been demonstrated to be further improved by the inclusion of nanoparticles with superior thermal conductivity, and the thermal conductivity of nanofluids rises with increasing nanoparticle content. As a result, LMs have received a lot of interest in fields such as thermal interface materials, heat dissipation chips, and, particularly, in fields of high-power sectors.

### 2.2. Chemical Properties

**Figure 3:**
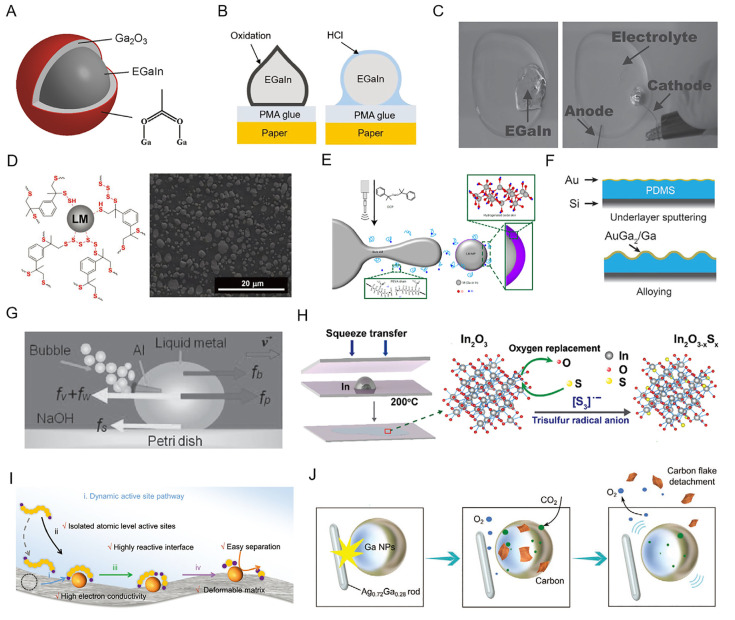
The fundamental chemical properties of LMs. (**A**) Illustration of the architecture of the oxidized liquid metal particle [42]. Copyright: (2014) American Chemical Society. (**B**) The schematic diagram of the liquid metal removed with HCL solution [45]. Copyright: (2018) WILEY-VCH. (**C**) Application of a reductive potential to the liquid metal removes the oxide [46]. Copyright: (2015) WILEY-VCH. (**D**) Scheme of the surface interactions between LM droplets with polysulfide loops (R-Sn-R) and thiol terminal groups (R-SH), and cross-sectional scanning electron microscope (SEM) image of the liquid-metal-embedded sulfur polymer [47]. Copyright: (2019) WILEY-VCH. (**E**) The process of hydrogen doping introduced by ultrasonication [48]. Copyright: (2021) Springer Nature. (**F**) Growth of biphasic gold–gallium thin films on a PDMS membrane [49]. Copyright: (2016) WILEY-VCH. (**G**) The Rehbinder effect between liquid metal alloy EGaIn or Galinstan and aluminum [50]. Copyright: (2015) WILEY-VCH. (**H**) Synthesis of 2D indium oxysulfide [53]. Copyright: (2021) The Royal Society of Chemistry. (**I**) Schematic illustration of the catalytic strategies using dynamic liquid metal electrocatalysts [51]. Copyright: (2022) WILEY-VCH. (**J**) Schematic illustration of the CO_2_ conversion process. The formation/detachment of the carbon flakes and the generation/escape of O_2_ are indicated [52]. Copyright: (2022) WILEY-VCH.

Pure LMs typically possess original smooth surfaces. However, once exposed to air due to oxidation, the surface of LMs spontaneously produce a solid oxide film, which substantially alters their surface behavior. A schematic illustration of the structure of an LM is shown in Figure 3A [42]. In ambient conditions, the LM always presents a core-shell structure. The shell is a gallium oxidation layer (Ga_2_O_3_) that protects the inner bulk LM core [43]. Liu’s team has demonstrated that the GaIn_10_-based liquid metal ink can generate outstanding wettability with nearly any desired materials, including epoxy resin board, glass, plastic, silica gel plate, paper, cotton, fabric, and more materials that have various levels of surface roughness [44]. Previous investigations have demonstrated that the amphoteric oxide layer on liquid metal particles could be eliminated in an acidic or alkaline solution. Guo et al. discovered that the EGaIn adhered to the polymethacrylates (PMA) glue could be removed with an HCl solution through this chemical reaction (Figure 3B) [45]. Additionally, the chemical connection between the PMA glue and EGaIn could be destroyed with the HCl solution in addition to removing the oxide layer. Furthermore, electrochemistry can also be used to reduce liquid metal oxides. As seen in Figure 3C, the oxide skin stabilizes the structure of an LM puddle dissolved in the electrolyte; however, when the metal is subjected to a reductive potential, the oxide is removed, and the metal beads up due to its high surface tension [46].

The LM’s chemical, interfacial, and rheological properties are greatly impacted by the solid structure of the oxide layer, which also adds more binding sites for functionalization. Strong forces between thiol and gallium caused the LM nanoparticles to lose some of their thick gallium oxide shells, which allowed for the exceptionally uniform dispersion of liquid metals in a bulk matrix (Figure 3D) [47]. Interestingly, the liquid-metal-embedded sulfur polymer exhibited amazing electrical conductivity even at low volume percentages of LM due to the uniform dispersion. Similar research has shown that ultrasonic hydrogen doping in the presence of a radical initiator causes the oxide skin to become highly conductive and flexible (Figure 3E) [48]. In addition, Ga can be alloyed with different metals. A new type of stretchable electronic conductors made of biphasic solid–liquid thin metal films was developed by Arthur Hirsch et al. [49]. The intermetallic compound AuGa_2_ was created when the gold film was entirely alloyed with the evaporated gallium, as seen in Figure 3F. After that, a heterogeneous film made of clusters of the solid intermetallic alloy AuGa_2_ and supercool liquid gallium was produced on the surface of the AuGa_2_ after the thermal evaporation of the liquid gallium. Additionally, the liquid metal alloy EGaIn or Galinstan and aluminum (Al) still exhibited the Rehbinder effect (Figure 3G) [50]. Al was securely adhered to the EGaIn because Al is susceptible to amalgamation with EGaIn. Al can be penetrated by EGaIn or Galinstan by removing the oxide skin of Al, which would further induce the redox reaction of Al in NaOH solution.

The surface oxidation layer is a naturally occurring two-dimensional (2D) material, and the capacity of liquid metals to catalyze reactions as well as the surface layering property both offer novel ways to create 2D materials. Figure 3H illustrates the transfer of the oxide skins (2D In_2_O_3_) from a molten indium metal droplet to the surface of 300 nm SiO_2_ silicon wafers. Then, in a solution of polysulfide radical anions, 2D In_2_O_3_ is converted into 2D In_2_O_3_-xSx. Sulfur atoms take the place of oxygen atoms during the process. In addition, the LM can also offer an active catalytic reaction platform. To facilitate the LiPS’s redox process, Qi et al. presented a unique dynamic electrocatalytic method using the liquid metal (Figure 3I) [51]. The major active catalytic core was made of Sn atoms that were dynamically dispersed throughout the liquid Ga matrix. Ga offers a particularly active environment, nevertheless, to preserve the long-term integrity of the catalytic system. By this mechanism, liquid-phase binary alloys show more options for use as electrocatalysts in high-specific-energy Li-S batteries. Additionally, green carbon capture and conversion can be achieved by employing LM mixed with Ga and the resilient intermetallic Ag-Ga structures (Figure 3J) [52]. A closed cyclic catalytic system was established to convert CO_2_ into solid carbon with an efficiency of 92%.

### 2.3. Biocompatibility

**Figure 4:**
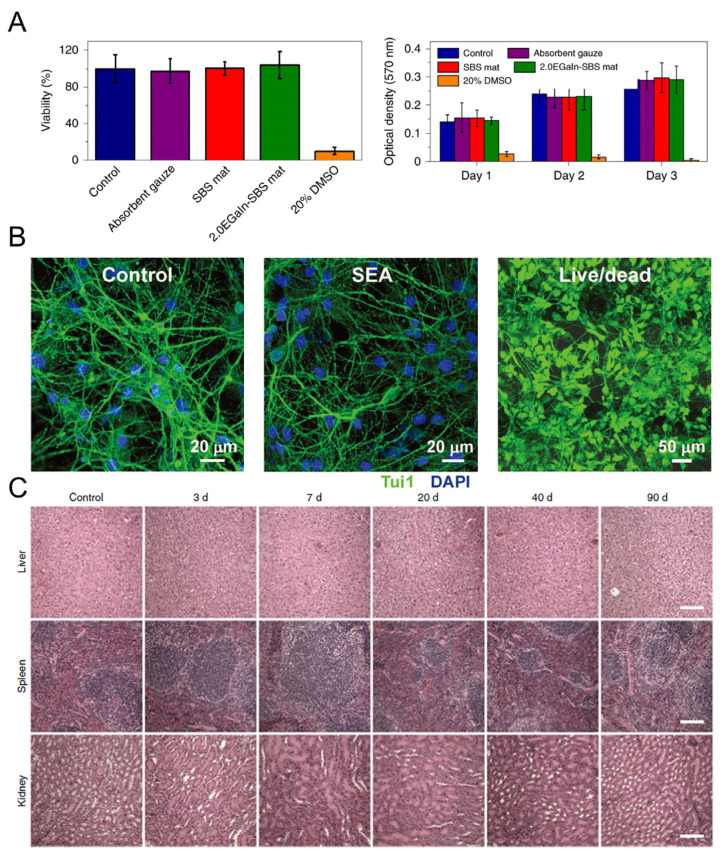
The biocompatibility of LMs. (**A**) The quantification of the live/dead-stained L-929 cells (**left**) and the absorption at 570 nm in MTT assay after 1–3 days of culturing [57]. Copyright: (2021) Springer Nature. (**B**) Fluorescent images of primary hippocampal neurons cultured on control tissue culture and liquid-metal-based neural electrodes at 14 days in vitro, and live/dead images of the hippocampal neurons on liquid-metal-based electrodes [59]. Copyright: (2015) WILEY-VCH. (**C**) Histology evaluation of the major organs (liver, spleen, and kidney) of LM-injected mice and control groups at different time points. Scale bars, 100 mm. d, days [60]. Copyright: (2015) Springer Nature.

Due to their high biocompatibility, typical low-melting alloys that contain Ga, In, Sn, and Bi elements have been used in a variety of biomedical and health technologies [54]. Among them, Ga and its compounds are attracting the most attention for research and clinical applications in the medical field [55]. The Ga-based electronics exhibited less tissue inflammation in the brain and ultra-softness on the skin in [56]. The low cytotoxicity of LM-based materials was verified by culturing L-929 cells for 3 days (Figure 4A) in [57]. The quantification of the live/dead-stained cells showed a high viability of almost 95%, and after a 3-day culturing, the absorption at 570 nm in the MTT assay increased obviously, which shows the feasibility of LM-based materials for biomedical uses. The biocompatibility of LM can be further enhanced by applying an alginate coating to delay the formation and release of harmful cations [58]. To verify the cytocompatibility and biocompatibility of LM, a 14-day in vitro experiment was conducted in [59]. The fluorescent images of the primary hippocampal neurons at 14 days and the live/dead labeling of hippocampal neurons at 7 days in vitro both further confirm the biocompatibility of LM (Figure 4B). In addition, biocompatibility was demonstrated by in vivo tests. A new liquid-metal-based nanoscale formulation for drug delivery to promote improved anticancer therapy was also disclosed by Lu et al. [60]. A necropsy on the mice was conducted, which noted that there was no obvious organ damage due to applying the LM (Figure 4C). No visible tissue damage was seen in tissues from the heart, brain, and muscle as well. As a result, the LM-NP/L showed no clear toxicity at the treatment dose, which was very positive for the use of LM-NP/L as a nanomedicine.

## 3. Fabrication Strategies of LMs for Epidermal Health Monitoring

As new-generation functional materials, low-melting liquid metal preparations are becoming increasingly significant. For stretchable electronics, liquid metals or their alloys provide a superior alternative for conductive and functional components due to their high conductivity and excellent fluidity. The high surface tension of alloys and their poor wettability with many substrates severely restrict the patterning of LMs, and for the mass production of LM-based electronics, there are still a lot of problems that need to be overcome, especially in the most-adopted printing technology. To create acceptable functional materials based on gallium or its alloys, numerous techniques have been developed, including microfluidics, surface modification, lowering surface tension via the additions of metals or elastomer materials, and printing. The application range of the low-melting liquid metals has been further enlarged to accommodate varied needs thanks to these technologies, which have not only allowed for the modification of their physical properties but also introduced a variety of new functionalities. This chapter summarizes the fabrication methods of functional LM materials for stretchable electronics.

### 3.1. Conductive Composites with Pure LM

**Figure 5:**
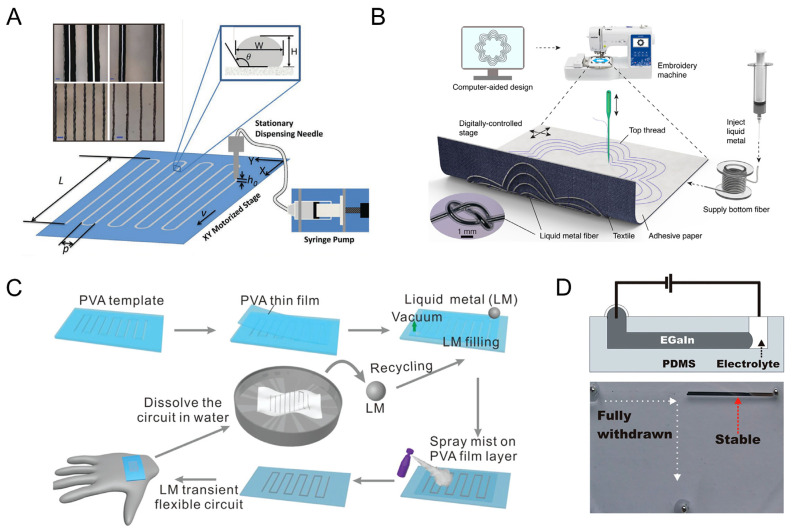
The fabrication strategies of conductive circuits with pure LM. (**A**) The direct writing system and the high-resolution wires directly written with LM [62]. Copyright: (2014) WILEY-VCH. (**B**) Illustration of the digital embroidery process using liquid metal fibers [63]. Copyright: (2022) Springer Nature. (**C**) Schematic illustration of the fabrication and recycling process of the room temperature LM-based PVA-encapsulated, recyclable, transient circuits [64]. Copyright: (2019) WILEY-VCH. (**D**) Schematic and photograph of the recapillarity-induced withdrawal of LM from microchannels [66]. Copyright: (2014) National Academy of Sciences.

Printing or injecting pure LM into a channel with a certain shape to create a functional circuit is a common technique for creating liquid metal microfluidic electronics. The atomically thin layer of liquid metal oxides exposed in the air can support a maximum surface stress of 0.5–0.6 N/m [61]. Boley et al. demonstrated a liquid metal alloy direct writing technology for making stretchy electronics on a small scale (Figure 5A) [62]. The liquid metal sticks to the flexible substrate due to the great adhesion of the oxide layer. The high surface tension of liquid metal restricts the resolution of the patterns to about 100 µm, even though this method validates the viability of one-step liquid metal patterning. By injecting Galinstan into perfluoroalkoxy alkane tubing, Lin and his team proved how to create liquid metal fibers that had excellent electrical and mechanical properties (Figure 5B) [63]. Then, by digitally embroidering the liquid metal threads into garments, electronic textile systems with near-field wireless power and communication capabilities were constructed. The article demonstrated the fabrics’ resistance to wear and tear even after being put through numerous washing and drying cycles as well as repeated stress and strain operations.

LM microchannels can be created in two-dimensional platforms by injecting liquid metal into prefabricated channels. Dickey et al. discovered that EGaIn presents the properties of elastic materials until applied with ~0.5 N/m surface stress, at which point the EGaIn can be pressed quickly (within 1s) to fill the channels. For microfluidic channels, a larger critical surface stress would be required, i.e., ~0.6 N/m, which is remarkably similar to the surface tension of EGaIn [61]. Figure 5C shows a vacuum-driven approach for filling a PVA microchannel with LM [64]. This method involves drilling two outlets at the microchannel’s ends, closing the holes with deposited LM droplets, and then degassing the entire apparatus. Due to the negative pressure that is created when the device is exposed to the atmosphere between the sealed microchannel and the environment, LMs are pumped into the PVA microchannel. As a result, it is easy to create transient circuits using LMs as the electronic circuit and PVA as the packaging material. Similar to this, Galinstan was injected into premade PDMS microchannels to create a wearable diaphragm pressure sensor [65]. It is worth noting that the removal of LM from the microchannels is difficult due to the adhesion of the oxide. By applying a reductive potential to the LM in the electrolyte, the capillary action can be reduced (also called “recapillarity”) with the removal of the surface oxide on LM [66] (Figure 5D). Then, the LM can withdraw in the direction of the cathode. In this way, the control of the capillary behavior of LM can be applied to the pumping of flow motion in the microchannel [67].

### 3.2. Strechable Composites with Micro- or Nano-Sized LM

**Figure 6:**
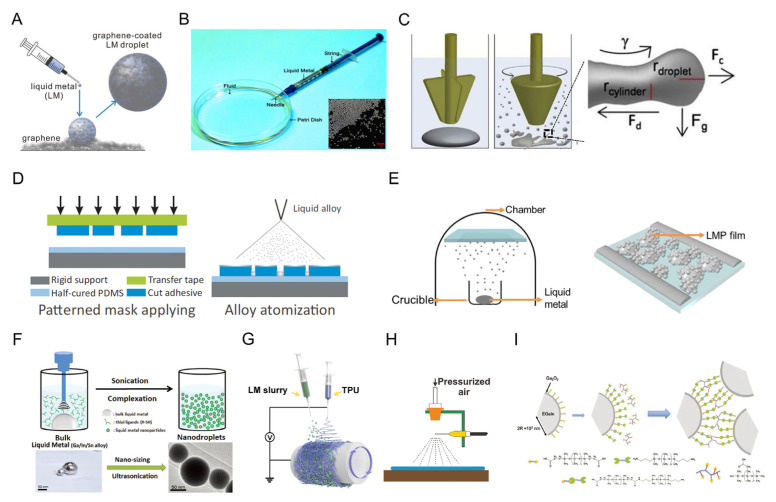
The fabrication strategies of LM-based conductive composites with size effect. (**A**) Schematic showing the generation process of graphene-coated liquid metal droplets [69]. Copyright: (2018) WILEY-VCH. (**B**) Device for injectable liquid metal droplets fabrication, and the inset image is the bottom view of liquid metal micro-droplets using a microscope [70]. Copyright: (2014) WILEY-VCH. (**C**) Schematic illustration of the SLICE process showing transformation of EGaIn into micro- and nanoparticles [42]. Copyright: (2014) American Chemical Society. (**D**) Manufacturing processes for liquid alloy microfluidic wireless power transfer [71]. Copyright: (2015) Springer Nature. (**E**) Thermal deposition process of liquid metal for liquid metal particle film and characterizations of liquid metal particle films under different preparation conditions [72]. (**F**) Schematic illustration of the preparation route for liquid metal nanodroplets [73]. Copyright: (2016) WILEY-VCH. (**G**) Schematic of electrospray of LM nanoparticles [74]. Copyright: (2022) WILEY-VCH. (**H**) Schematic illustration of air spray printing of LM slurry [75]. Copyright: (2017) WILEY-VCH. (**I**) Schematic illustration of the preparation of the surface-modified EGaIn nanoparticle elastomer [77]. Copyright: (2021) WILEY-VCH.

Micro- and nano-sized LM droplets are fabricated to realize easier access to the application of LM [68]. The size of LM can be regulated by the fabrication methods, including inkjet directly, inkjet in solution, mechanical stirring, atomization process, ultrasonic treatment, thermal evaporation, etc. Monodisperse and spherical microdroplets with a diameter of roughly 3 mm were fabricated by extruding and rolling LM droplets on graphene sheets (Figure 6A) in [69]. An outer layer of densely packed graphene sheets was created because of the quick uptake of the graphene sheets by the LM droplets’ surface. Based on the concepts of flow injection and self-breakage, Yu and his collaborators present a procedure for injecting liquid metals into matching solutions to produce liquid metal microparticles with a diameter of ~300 μm (Figure 6B) [70]. A lot of liquid metal droplets are immediately created as the plunger is pressed. This results from a balance between the surrounding solution’s shear force and the liquid metal’s surface tension. This study paves the way for the mass production of liquid metal microdroplets and particles in a very straightforward manner.

Simple mechanical stirring or blending with other functional materials can likewise distribute liquid metal bulks into liquid metal micro–nano droplets. Low melting point metal alloys are mechanically stirred under fluid flow to produce tiny particles (Figure 6C) [42]. By vigorously swirling, the oxide layer can be broken, allowing more and more metals to oxidize, creating a uniformly spread mixture of the liquid metal and its oxide. By shearing in an acidic liquid medium, they were able to create smooth liquid core–shell particles ranging in size from 6.4 nm to over 10 µm.

The atomization of LM enables its applications in patterning on multi-substrates via spray deposition. Figure 6D illustrates the production steps of an atomization-patterning liquid alloy both for liquid alloy patterning and further rigid components’ hybrid integration [71]. This is also a universal fabrication approach for producing high-quality patterns of LM with improved shape definition. In cases of physical vapor deposition, Galinstan can be transited from the condensed phase (Galinstan bulk liquid) to the vapor phase (Ga, In, and Sn atoms) and then back to a thin condensed film on the targeted objects [72]. Li and colleagues effectively created LM particle films using secondary thermal evaporation without reducing the surface tension in addition to managing the behavior of liquid metals with microchannels (Figure 6E) [72]. After being exposed to air to generate an oxide layer, the sample of liquid metal with one layer is once more coated with LM. On top of the oxide layer, the second layer of LM e-cells is created to realize the conductivity of the LM-based line.

In addition, a straightforward ultrasonication method in the presence of organic solvents may be used to turn LM into nanoparticles to produce numerous functionalities. The walls of a Petri dish are struck by LM during the process of ultrasound. This dispersion is mostly caused by the considerable energy that the LM gained during the collision with the wall. When the surface tension of LM is unable to contain the energy, the LM droplets break down into smaller nanoparticles to decrease the energy. Most of this ultrasonic dispersion occurs in liquid environments where emulsifiers are present. As seen in Figure 6F [73], cavitation is created by applying an ultrasonic probe to a mixture of liquid metal bulks, thiols, and ethanol. This causes localized extremes in pressure and temperature to occur within a very short amount of time. Under the influence of oscillating shear force, the LM body that was supported by the oxidation layer in this instance is quickly split into several LM nanoparticles. After this, the LM nanoparticles with the ultrasonic treatment could be composited into elastomers using electrospraying [74], air spraying [75], and so forth (Figure 6G,H).

In the meantime, the surface oxidation layer of LM serves as an ideal stage for the functionalization and polymerization of the LM nanoparticles [76]. Thiolated ligands can easily and robustly assemble on the surface of the developing LM nanoparticles [77]. Furthermore, the spherical LM nanoparticles are stable due to the defense provided by the thiolated ligands and the rapid oxidation of the surface. Additionally, it is simple to add certain functional radical groups to liquid metal nanoparticles and encapsulate them in a nanocapsule. As seen in Figure 6I, carboxylic acid-ended polydimethylsiloxane was used to modify the surface of EGaIn nanoparticles. It is possible to create dense EGaIn nanoparticles in a PDMS matrix with efficient thermal transfer by crosslinking with the surface-modified EGaIn nanoparticles. In addition, the dipole–dipole interactions between LM and elastomers were studied to enhance their electrical and mechanical stability [78]. A coaxial wet-spinning method was proposed to continuously create super-elastic EGaIn sheath–core microfibers with both high and ultra-stable conductance to address the issue of leakage of conductive materials in liquid metal fibers. The core of the microfiber is made of a combination of the same fluoroelastomer and percolated EGaIn nanoparticles, while the sheath is made of a double-network fluoroelastomer with excellent elasticity.

### 3.3. Surface Tension Reduction through the Mixture

**Figure 7:**
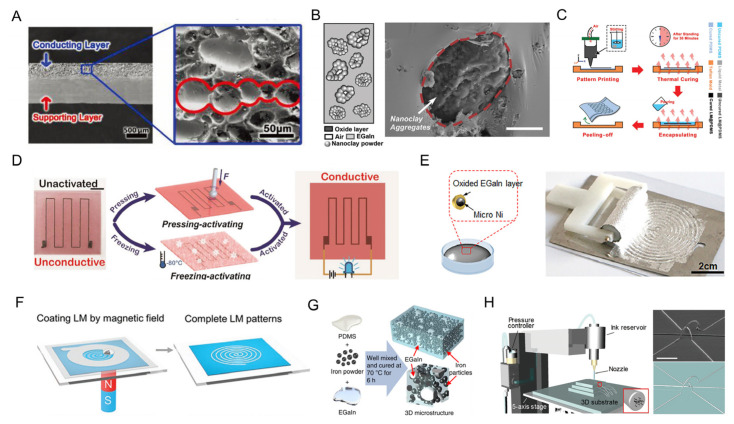
The surface tension reduction of LM via mixture. (**A**) Scanning electron microscope (SEM) cross-sectional view of the prepared LM-based composite elastic conductor with the desired 3D-Calabash Bunch conductive network structure in PDMS matrix [32]. Copyright: (2018) WILEY-VCH. (**B**) Schematic and the cross-sectional SEM of conductive nanoclay (scale bar: 20 mm) [79]. Copyright: (2021) The Royal Society of Chemistry. (**C**) Schematic depiction of the fabrication process for creating LM@PDMS stretchable, wearable electrically driven heaters [80]. Copyright: (2019) WILEY-VCH. (**D**) Activation of the printed electrical route through pressing and freezing [83]. Copyright: (2019) WILEY-VCH. (**E**) Schematic of Ni-GaIn amalgam, and the digital photograph for screen printing by a rolling brush [85]. Copyright: (2018) WILEY-VCH. (**F**) Schematic of high-resolution patterning of magnetic microparticle-doped LM via magnetic field control [86]. Copyright: (2019) WILEY-VCH. (**G**) Production of the liquid metal-filled magnetorheological elastomer [89]. Copyright: (2019) Springer Nature. (**H**) Schematic of the 3D direct printing system of CNT/LM composites (left), and the SEM images of 3D-printed CNT/LM composites [88]. Scale bars, 100 μm. Copyright: (2019) American Chemical Society.

The high surface tension of LM can be reduced through a mixture of elastomers or other metal particles. The final composites would possess high viscosity that facilitates the processability of LM-based conductive networks. On the one hand, an elastic conductor with good conductivity and stretchability is created using highly conductive and flexibly deformable LM fillers. LM can be blended directly with polymers in addition to combining with metal to create elastic conductors. Galinstan and PDMS were combined and stirred to create an LM-based composite elastic conductor with a 3D-Calabash Bunch conductive network structure, as shown in Figure 7A [32]. Based on this kind of LM-based elastomer, conductive circuits can be fabricated by using a variety of techniques, including stamp printing [79], inkjet printing [80], etc. In [79], a nanoclay was introduced into an LM system to prepare a low-fluid and highly adhesive composite. The nanoclay clumps were gradually wrapped with the Ga_2_O_3_ layer and distributed in the LM (Figure 7B). This composite was also an ideal direct-printable ink for in situ fast stamp patterning. In addition, Wang et al. demonstrated that increasing the proportion of liquid metal in printing ink can result in a printing method with excellent resolution (Figure 7C) [81]. The cause is that as the LM filling fraction rises, internal friction between these two liquid phases develops and works against the flow of the LM@PDMS composite.

In some cases, mechanical stress was required to induce the rupture of LM particles and form conductive networks. The relationship between the LM particles and different elastomers was systematically studied in [82]. Zhou et al. showed that LM–silicone ink, a concentrated mixture of LM microdroplets and silicone elastomer, demonstrates originally electrically isolated but outstanding printability with excellent resolution for direct printing (Figure 7D) [83]. Despite it being originally nonconductive, LM–silicone can be made conductive through pressing or freezing. The activated composite would possess excellent conductivity and a significant electrical response to strain.

LM mixtures that are fabricated via the addition of other rigid metal particles, such as copper, gold, silver, and nickel (Ni), exhibit low viscosity and are easily processible as well [84]. Further to this, the addition of nickel or iron can further introduce the magnetic properties that enable the precise control of LM wire, high-resolution patterns, remote self-healing circuits, etc. [26]. The Ni particles would be wrapped with a Ga_2_O_3_ layer as they are mixed with the EGaIn, as in [85] (Figure 7E). This mixing process would accelerate the oxidation of LM and finally lower the surface tension of LM. LM-based stretchable coils and circuits can be fabricated by directly screen printing of LM on Ecoflex. In addition, the high-resolution of LM patterns was developed by introducing magnetic microparticles into the LM in [86] (Figure 7F). A shadow mask was applied onto the targeted hydrogel, under which a magnet was used for attracting and aggregating the magnetic microparticle-doped LM onto the shadow mask. The proposed LM-based circuit would be fabricated with the removal of the shadow mask. The combination of LM, elastomer, and rigid metal particles was developed to realize a multifunctional composite. A liquid-metal-filled magnetorheological PDMS with iron microparticles and LM is reported in [82] (Figure 7G). This composite achieved a novel positive piezoconductive effect that decreases the electrical resistance dramatically upon any deformations, presenting promising potentials for flexible sensors and responsive thermal interfaces.

It is challenging to combine printing since the melting points of conductive metal and non-conductive ink in conventional 3D printing differ by hundreds or even thousands of degrees Celsius at the same time. The current 3D printing of high melting point metal has stringent requirements for processing conditions and the associated machinery. The drag adhesion effect of a liquid metal with a low melting point can compensate for this. The viability of printing and assembling functional devices by using low-melting LM inks was proved completely in [87]. The necessary material and processing for stretchable 3D interconnections on soft substrates with high resolutions are then introduced by Park et al. (Figure 7H) [88]. By adding carbon nanotubes to the liquid metal, the low mechanical strength of the liquid metal is overcome, improving the mechanical strength of the composite. The minimal diameters for the composites, which can be 3D printed, are around 5 μm.

### 3.4. LM–Elastomer Interface Enhancements

The high surface tension of LM makes it difficult to spread onto elastomer surfaces. The interfacial interactions between LM and elastomer have attracted huge attention recently due to the requirement for more stable electrical components. Main strategies have been developed for the enhancement of LM–elastomer interface interactions, i.e., hydrogen bond introduction, self-adaptable scaffold, and alloying process introduction.

**Figure 8:**
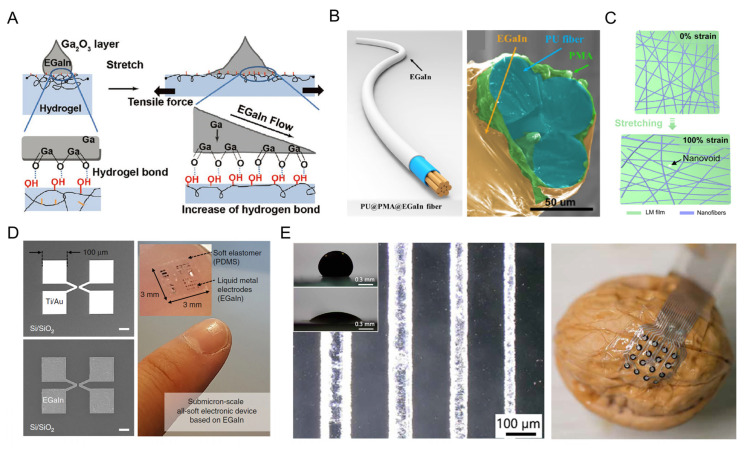
Enhancement strategies for LM–elastomer interfaces. (**A**) Schematic of the interactions between Ga2O3 layer with -OH groups on hydrogel upon stretching [91]. Copyright: (2020) Wiley-VCH. (**B**) Schematic and cross-section SEM of PPE fiber coated with PMA and LM [96]. Copyright: (2020) American Chemical Society. (**C**) Schematic of LM-nanofiber scaffold interface under stretching [74]. Copyright: (2022) Wiley-VCH. (**D**) Micro-morphologies of patterned Au (**left top**) and EGaIn on Au (**left bottom**). Scale bar: 40 μm. The encapsulation of the EGaIn structures on a fingertip [94]. Copyright: (2020) Springer Nature. (**E**) Optical microscopy images of the LM line array (**left**) with contact angle images of the LM on SEBS and copper (**left inset**), and digital photography of the conformal attachment of an LM-based electrode array [95]. Copyright: (2022) AAAS.

Hydroxyl groups’ (−OH) introduction is the key to enabling the interactions with the Ga_2_O_3_ layer outside the LM [90]. Hydrogels provide numerous hydroxyl groups associated with water and also control the stretchability of a hydrogel [91] (Figure 8A). The LM can be directly spread onto the chemically cross-linked hydrogel and patterned into various electrodes and circuits. An autonomous surface reconciliation of LM was observed on the hydrogel surface even at a stain of 1500%. The ultra-low electrical resistance and resistance variation of LM on hydrogel verified its potentials in microelectronics. In addition, polymethacrylate (PMA) is another polymer that shows an excellent affinity for LM due to the hydrogen bonding between the aliphatic groups of PMA and the Ga_2_O_3_ oxidation layer (Figure 8B). Underlaying with polyurethane (PU), LM conductive fibers were fabricated for stretchable conductors and wearable sensors.

The structure design of LM–elastomer interfaces is another strategy for enhanced interactions. The mechanism lies in the formation of the Laplace capillary force around each microvoid of elastomer scaffolds (Figure 8C) [74]. This force would attract the LM film and achieve a mechanical balance upon the gravitational force. The superiority of this strategy is based on the structure design without other material modifications. As a result, a highly adaptable interface would be constructed for robust electrical performance under dynamic deformations. A similar phenomenon can be observed in the LM@SBS structure and LM@TPU structure [57,92].

A room-temperature interfacial alloying process occurred as an LM contacted other metals such as Au and Ag. The new compositions of AuGa_2_ clusters, AgNP-Ga-In clusters and the supercool LM surrounding them were fabricated correspondingly in [49,93]. This alloying process overcomes the cohesive forces by allowing LM to diffuse and form a continuous conductive film, which further enables the high-resolution fabrication of electronic tattoos, 3D hydrographic transfer films, etc. A sub-micron-scale and all-soft LM-based electrode was developed by applying the hybrid lithography process in [94]. Between the LM and PMMA, a deposited Ti/Au layer was fabricated to enhance the adhesion and wetting characteristics of LM (Figure 8D). The overall electronic devices can be encapsulated with a size of only 3 mm × 3 mm on the fingernail. In addition, the alloying of LM is further exemplified by the interaction between copper (Cu) and LM. Compared with the LM on pure SEBS, the contact angle of LM on Cu decreased to 30° from 130° to verify the enhanced wettability through an interfacial alloying reaction in [95]. Thus, the LM line resolution could be reduced to ~50 μm. The Cu pattern could be selectively patterned with LM and construct a 4 × 4 electrodes array for conformal attachments to internal organs and a walnut with a complex surface morphology.

## 4. Applications

### 4.1. Electrode for Biomedical Signal Collections

**Figure 9:**
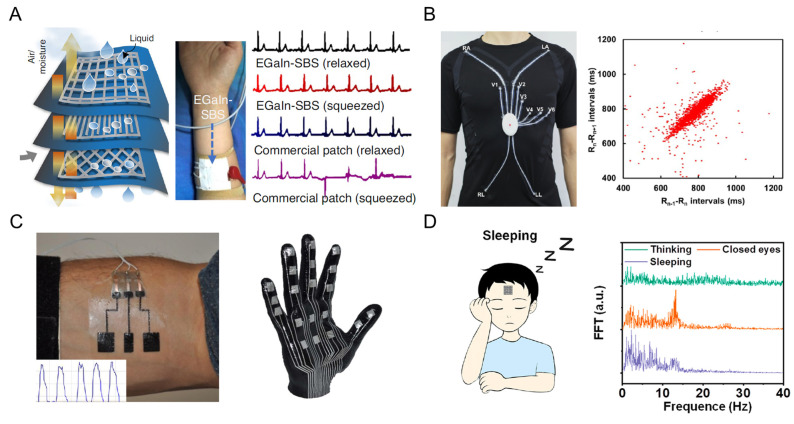
The LM-based electrode for real-time physiological signal collection. (**A**) Schematic illustration of LM-based monolithic stretchable electronics in which the top layer is the electrode for ECG monitoring [57]. Copyright: (2021) Springer Nature. (**B**) Image of LM-based 12-lead ECG monitoring system on a knitted T-shirt (**left**) and the Lorenz plot of ECG under the sleeping condition of the volunteer [100]. Copyright: (2022) American Chemical Society. (**C**) The LM electronic tattoo for EMG signal acquisition on a forearm (**left**) and capacitive sensor mesh on a 3D hand model. Copyright: (2018) American Chemical Society [98]. (**D**) Schematic diagram of the real-time EEG recording in the mental state of sleeping, and the frequency distribution of EEG with fast Fourier transform [99]. Copyright: (2022) American Chemical Society.

The intrinsic function of LM-based materials for epidermal healthcare is conduction, through which devices can collect physiological signals based on electrical potential differences and serve as conductive wires for the connection of various sensors [91]. ECG is a painless and non-invasive strategy for individual heart performance and a standard process in modern cardiovascular medicine [97], using electrodes on the skin to monitor the electrical changes of the cardiac muscle depolarization and repolarization during the cardiac cycle (commonly known as the heartbeat). An LM electrode was directly attached to the human epidermis for reliable ECG data collection (Figure 9A). Due to the superelasticity of LM, the low-noise ECG signals were maintained robust when the electrode was stretched or compressed, which was unachievable for commercial electrode patches [57]. For a large area of electrical circuits, a 12-lead ECG circuit was printed on a knitted T-shirt for cardiac activity monitoring (Figure 9B). The LM circuits enable stable ECG signal collection during sitting, lying, and walking. In addition, the R-R intervals under sleeping conditions were calculated and plotted through the Lorenz strategy to diagnose the health condition of the volunteer.

In addition to ECG monitoring, LM-based electrodes can be applied in EMG and EEG signal collection. An electronic tattoo was demonstrated with Ag-In-Ga trances that present high conductivity and conformability to nondevelopable 3D surfaces (Figure 9C) in [98]. This tattoo can be easily placed over a human forearm by hydroprinting to collect EMG signals, which can be subsequently applied as a human–machine interaction to control a robot hand prosthetic. In addition, precise EEG recording is always a challenge due to its weak signal. A kirigami-structured LM paper that is conductor-exposing and ultrathin was fabricated and attached to a human forehead for real-time EEG signal recording (Figure 9D) in [99]. Three different frequency bands of EEG in three mental states were differentiated with fast Fourier transform, i.e., beta wave (~24 Hz), alpha wave (~12 Hz), and delta wave (~24 Hz) for thinking, closing eyes, and sleeping, respectively.

### 4.2. Interconnectors for Functional Circuits

**Figure 10:**
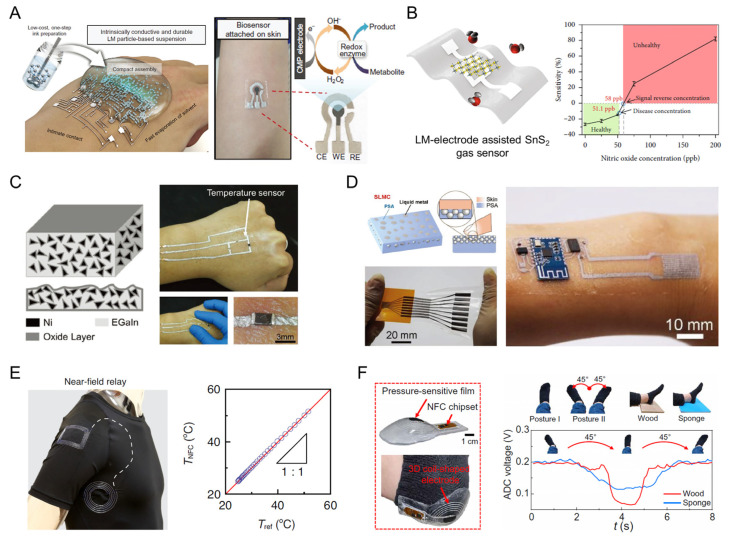
The multifunctional epidermal healthcare biosensors that are enabled with LM-based conductors. (**A**) Schematic of Pt-decorated CNT-attached LM particles (CMP) for on-skin e-tattoo (**left**). The photograph of CMP-based biosensor for sweat chemistry [102]. Copyright: (2022) WILEY-VCH. (**B**) Schematic illustration of LM electrode-assisted gas sensor with SnS_2_ (**left**). The sensitivity of gas sensors regarding NO gas concentrations [103]. Copyright: (2021) AAAS. (**C**) Schematic illustration of the structure of nickel–EGaIn conductive materials (**left**), and the photographs of the temperature monitoring circuit connected with LM conductors [104]. Copyright: (2019) WILEY-VCH. (**D**) Schematic of the structure of the intrinsically sticky LM conductor and photographs of its applications in stretchable conductive interfaces [105]. Copyright: (2022) WILEY-VCH. (**E**) Battery-free textile thermal monitoring system with LM fiber patterns for battery-free thermal monitoring and calibration of measured temperature with a commercial reference thermometer [63]. Copyright: (2022) Springer Nature. (**F**) Photographs of the LM-based heel-shaped wireless battery-free pressure sensor and pressure signals during various foot postures [106]. Copyright: (2021) AAAS.

Serving as stretchable circuits and connectors, LM composites exhibit exceptional electrical robustness and high conductivity upon on-skin deformation, making them a potential candidate for a wearable biosensor. Skin-attachable biosensors are of significance for healthcare monitoring and disease diagnoses such as diabetes and rehabilitation [101]. Carbon nanotubes (CNTs) can form an electrostatic interaction with LM particles with the help of negatively charged polyelectrolytes (poly(sodium4-styrene sulfonate) (PSS)) (Figure 10A) [102]. Based on this, an intrinsic electrical conductivity was imposed for carbon nanotubes on LM particles (CMP) whilst coated on human skin. After functionalization with glucose oxidase, alcohol oxidase, and lactate oxidase, the CMP-based working electrode realized the measurement of glucose, ethanol, and lactate. Additionally, the respiratory activity monitoring of humans plays a critical role in non-invasive medicine. An LM electrode can be applied to an epidermal respiration sensor for monitoring the level of relative humidity and the concentration of NO gas exhaled by humans (Figure 10B) [103]. Through this, different respiratory patterns were distinguished based on the water amount and potential lung diseases (e.g., asthma) can be diagnosed timely.

Then, the LM-based circuit would facilitate multifunctional sensors when equipped with various rigid chips, e.g., real-time temperature monitoring, near-field communication (NFC)/radio frequency identification devices (RFID), and interconnectors with a PCB board. To reduce the surface tension and improve the wettability for more processibility, a nickel and EGaIn mixture was developed to be printed as an electronic tattoo on human skin (Figure 10C) in [104]. When connected with a temperature sensor chip, the Ni-EGaIn conductor could realize a stable conductivity upon the movement of the wrist and real-time temperature monitoring of the epidermal research area. Moreover, an LM-based wireless electronic tattoo that is sticky and intrinsically conductive was designed for a more complex device fabrication (Figure 10D) in [105]. The wrist movement monitoring device was realized through a microcontroller, resistance, Bluetooth, and a strain sensor that were connected with a sticky LM conductor (SLMC), which could form both electrical and mechanical connections once the rigid chipsets or soft tissues contacted it.

Moreover, an LM-fiber-based textile electronic system was designed for near-field powering and communication [64], where LM fibers worked as an NFC relay with an inductor and a thermal sensor embroidered into the textile underarm (Figure 10E) in [63]. The axillary temperature can be continuously and correctively monitored as compared with a thermometer with a range from 25 to 55 °C. It is worth noting that the data stream would stay stable even if the textile operated in a wet environment, thanks to the water-proof encapsulation of elastomer. In addition, another LM-based battery-free sensor was illustrated for the bedsore healthcare system (Figure 10F) in [106]. The fluidity and conductivity make it possible to form a 3D hemisphere-shaped wireless power transmission component to connect with a pressure-sensitive sensor for the identification of different heel laying positions on the different hardness of the floors. Additionally, with the NFC reader, continuous pressure measurements were collected stably due to the conductive robustness of LM under dynamic deformations.

### 4.3. Mechanical Sensors

**Figure 11:**
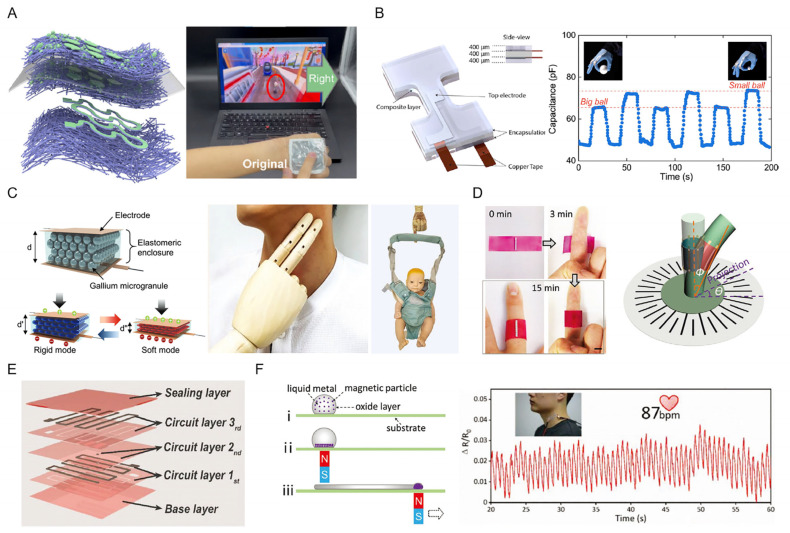
Epidermal mechanical sensors assisted by capacity and resistance. (**A**) Schematic illustration of LM electrode-based human–machine interactive system and photographs of on-skin pressure sensor array that shows potential for perception enhancement for burned skin [74]. Copyright: (2022) WILEY-VCH. (**B**) Schematic of LM dielectric layer for the capacitive sensor to evaluate strain deformations (**left**). The capacitance variation of grasping balls with different sizes [108]. Copyright: (2019) American Chemical Society. (**C**) Schematic illustration of the sensing mechanism for rigid and soft mode (**left**), and the optimal images for measurement of subtle pressure and large loads induced by carotid artery pulse and human weight, respectively [35]. Copyright: (2022) WILEY-VCH. (**D**) The photograph of the morphing process of the LM-based hydrogel sensor (**left**) and the schematic of it for bending and azimuth angle measurement of a rubber tube [107]. Copyright: (2021) WILEY-VCH. (**E**) Schematic of LM-based multilayer soft electronics [83]. Copyright: (2019) WILEY-VCH. (**F**) Schematic illustration of LM patterning operation step (**left**). The photograph and plot for the monitoring of carotid arterial pressure (**right**) [80]. Copyright: (2019) WILEY-VCH.

Through the structure design of LM-based electronics, mechanical sensors can be fabricated based on the mechanisms of capacity, resistance, piezoelectricity, triboelectricity, etc. When fabricated as a capacitive sensor, LM could serve as the electrode with high conductivity or the diametric layer with a non-conductive and high-dielectric constant. An LM-based highly robust electrode was fabricated with electrospinning and electrospray, after which a capacitive sensor array, named an adaptive human–machine interactive system, was designed via the particle activation of the original composite (Figure 11A) in [74]. This array could be attached to the back of a hand to input commands to a computer. Additionally, this LM-based capacitive sensor remained functional even under stretching. This e-skin exhibits promising potentials for fabricating biocompatible epidermal pressure sensors for perception enhancement of burned skin. On the other hand, LM-based composites were developed for high-dielectric layers in a capacitive sensor. Through independently controllable LM droplet size and volume loading in elastomers, the dielectric constant can be coordinated and reach a high relative permittivity of 60, which is 16 times that of unfilled elastomers (Figure 11B). An all-soft capacitive sensor with tunable sensitivity was demonstrated for the gesture quantification of the proximal interphalangeal. In addition, the solid–liquid phase transition of the gallium microgranule-based dielectric layer could facilitate a wide range and high sensitivity of the sensors (Figure 11C) [35]. This temperature-dependent rigid-soft mode conversion provides a 97% lower minimum and 262% higher detectable pressure, compared with the detection range of human skin. As a result, subtle blood pulsation and body weight can be detected simultaneously.

Resistance-based sensors are also preferred for epidermal sensing among LM-based electronics [86]. The structure of LM strips is commonly applied to amplify resistance change as a function of strain or pressure. A hydrogel-based soft electrode via the stencil printing of LM presents a self-shaping ability that can actively deform into 3D configurations of objects, such as human fingers (Figure 11D) [107]. By simultaneously integrating eight sensing units (LM strips), the sensor could monitor the direction and amplitude of bending motion. In addition, it is possible to fabricate the strain sensor as a multi-layer structure for more potential applications. The mixture of LM microdroplets and silicone elastomer, named LM–silicone ink, was developed to produce stretchable electronics via direct ink writing (Figure 11E) [83]. A multilayer of a soft strain sensor was printed directly, possessing exceptional repeatability and ideal linearity. Another geometrical strain sensor, with a cylindrical shape, was printed and embedded in gloves to realize hand gesture recognition. Furthermore, the fundamental mechanism of the resistance increment upon pressure is based on the cross-section reduction in this process. A continuous conductive line could be patterned onto elastomer or other super-metallophobic substrates via magnetic field control on an LM and magnetic microparticle composite (Figure 11F) in [80]. Encapsulated by the biocompatible Ecoflex, this LM line could achieve a sensitivity of 0.37 kPa^−1^ and be used for the real-time measurement of carotid arterial pressures.

Mechanical sensors based on other mechanisms, such as electromagnetic induction, electrical induction, triboelectricity, and piezoconductivity, were also applied in flexible on-skin healthcare, where the LM mainly serves as a conductive element. A liquid metal spiral coil was developed to harvest electrical energy under a permanent magnetic field, forming a self-powered stretchable sensor (Figure 12A) in [109]. When it comes to mechanical deformation, the energy conversion occurs with an output of 2 mA short-circuit current based on Faraday’s law of electromagnetic induction. Hand trembles (e.g., of Parkinson’s disease) can be successfully detected in terms of amplitude and speed characteristics, which exhibits promising potentials in the real-time detection of hand tremors or deviant finger movements. Alternatively, electrical inductive sensors are based on the inductance variation in response to changes in geometric parameters. ecause induction is usually related to the length, diameter, and number of coils, a deformable conductive fiber that consists of hollow thermoplastic polyurethane fibers with LM inside was developed and manufactured into a helical coil to monitor finger gestures (Figure 12B) in [110]. The inductance increased with the increase in the bending angle.

In addition, electromagnetic interference shielding effectiveness (SE) is a significant parameter for electromagnetic pollution prevention. A multifunctional electromagnetic film was developed with a magnetic LM droplet-filled elastomer (Figure 12C) in [111]. Upon elongation, the SE of this film significantly increases with a linear response. Therefore, this layer was designed as an off/on switchable component in a wearable strain sensor on a human elbow, combined with another functional layer of flexible micro-antennas. The remote electromagnetic source would pass through this shielding film as unstretched and be blocked as the elbow bends.

**Figure 12:**
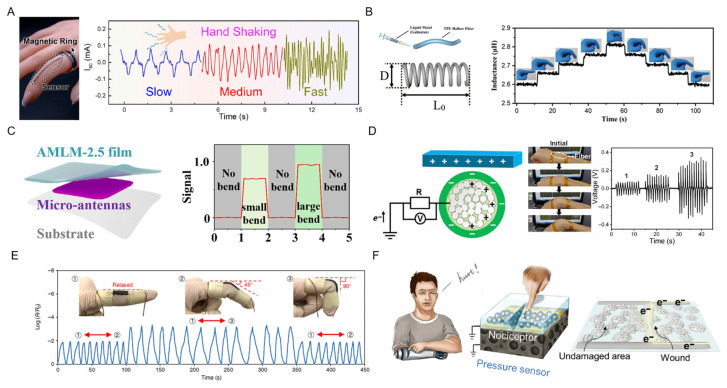
Mechanical sensors based on other mechanisms such as electromagnetic induction, electromagnetic shielding, electrical induction, triboelectricity, and piezoconductivity. (**A**) Photograph of the sensor on a finger (**left**). The output of sensor under hand trembling at slow, medium, and fast speed (**right**) [109]. Copyright: (2022) Elsevier. (**B**) Schematic of deformable conductive fiber fabricated from LM embedded into hollow elastomer tubes, and the design parameters of the helical inductive sensor (**left**). The inductance variation with the bending of the finger [110]. Copyright: (2021) Royal Society of Chemistry. (**C**) Schematic of the wearable strain sensor on an elbow (**left**), and signals induced by the micro-antennas layer responding to the deformation of the elbow [111]. Copyright: (2022) Elsevier. (**D**) The mechanism of the triboelectric sensor (**left**) and photograph when it was attached to a human wrist accompanied with corresponding voltage output [78]. Copyright: (2021) AAAS. (**E**) The application of the LM-based piezoconductive elastomer for finger bending detection [89]. Copyright: (2019) Springer Nature. (**F**) Schematic of the damage-detection sensor with the injury protection mechanism [72]. Copyright: (2022) WILEY-VCH.

Triboelectric nanogenerators and sensors are based on the contact electrification and electrostatic induction effect [112]. A stain-insensitive intrinsically conductive LM sheath–core microfiber was developed using a coaxial wet-spinning process (Figure 12D) [78]. For the fabrication of a single-electrode mode sensor, the LM core needs to be connected to the ground. Then, the electrification occurs at the interface of the elastomer sheath (electronegative surface) and human skin (electropositive surface) where they contact each other, after which the electron movements in the LM core are induced with the separation of these two surfaces. We note that this triboelectric self-powered sensor, attached to a human wrist, could monitor real-time wrist movement.

The mechanism of positive piezoconductivity for LM-based composites was proposed through an LM-filled magnetorheological elastomer, which consists of LM microdroplets and magnetic microparticles (Figure 12E) in [89]. It is unique that the resistance of the sensor remains high in a relaxed state and drops sharply when it suffers from any mechanical deformations. As a result, the movement of human joints can be detected with excellent stability for wearable devices.

Other novel structure designs of LM components pave pathways for the detection of algesthesia by taking advantage of its fluidity and self-healing properties [34]. More importantly, a LM-based damage-detection sensor provides a potential for a skin-mimic after-injury protection mechanism when it is controlled by an artificial synapse (Figure 12F) in [72]. In this sensor, the LM particle film fabricated with physical vapor deposition was the key component for damage detection. The mechanism relies on the resistance decrease from the rupturing of LM particles at the damage point.

### 4.4. Thermal Management

Thermal management is becoming increasingly important to meet the basic requirement of human thermal comfort and other medical healthcare strategies [113]. For on-skin electronics, two kinds of devices are dominantly attractive in research, i.e., heat dissipation and thermal therapy devices. Through programming the LM microstructure, oriented LM droplets in elastomer were fabricated as a soft heat sink (Figure 13A) in [77]. Compared with the pure elastomer region, the oriented-LM regions present a lower maximum temperature (40 °C) due to their higher thermal conductivity. In addition, a Janus structure of LM-based composite was developed via the self-assembly density deposition of LM droplets (Figure 13B) in [114]. The thermal profile of the LM-rich side was 9 times higher than that of polymer-rich insulating side, i.e., 0.525 and 0.0606 W m^−1^ K^−1^, respectively. This unique characteristic of thermal conductivity difference shows its potential for on-body thermal management textiles or energy-saving buildings.

**Figure 13:**
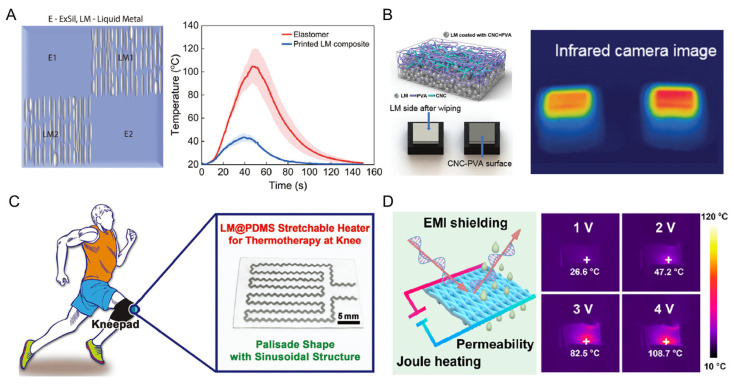
The LM-based electronics for epidermal thermal management. (**A**) Schematic of the multilateral printed heat sink with four regions (**left**), and the temperature of the LEDs on four regions (**right**) [41]. Copyright: (2022) WILEY-VCH. (**B**) Schematic of the LM-based Janus film for thermal management (**left**), and the infrared image of their thermal management [114]. Copyright: (2019) The Royal Society of Chemistry. (**C**) Schematic of the LM@PDMS heater in a palisade shape for articulatio genus thermal treatment [81]. Copyright: (2019) WILEY-VCH. (**D**) Schematic illustration of the electromagnetic interference shielding and joule heating (**left**). The joule heating performances of the device under different voltages [116]. Copyright: (2020) American Chemical Society.

Continuous thermal therapy and warmth retention are the most common strategies for the applications of conformal and conductive composites on skin based on joule heating [115]. LM-based conductors can exhibit rapid responses in heating upon applied voltage due to their intrinsically exceptional conductivity and flexibility. An LM@PDMS composite was patterned in a serpentine geometry as a stretchable thermal heater using direct printing (Figure 13C) [81]. The dynamic conductive stability of LM@PDMS meets the requirements of wearable thermotherapy in the scenario of doing exercise. Thus, a LM@PDMS heater in a palisade shape with an optimal sinusoidal structure was prepared and applied to the thermal treatment of the articulatio genus. In addition to the joule heating, the high conductivity also contributes to the enhancement of the absorption and reflection of electromagnetic (EM) waves, which may eventually promote the shielding effectiveness of the on-skin electronics (Figure 13D) [116]. The EM interference shielding and rapid joule heating can be simultaneously achieved for human body protection and therapies.

### 4.5. Other Biomedical and Sustainable Applications

Other aspects of LM-based applications that are important in epidermal healthcare electronics have also been explored, including biosensing and energy supplying, epidermal micro-environment care, and epidermal protection (wound healing and anti-microbial applications). A silver–LM island–bridge structure was constructed with the advantages of both mechanical and electrical resiliency under dynamic deformations (Figure 14A) in [117]. This structure presents reliable electrochemical stability for the sensing of potassium hexacyanoferrate (III) after cyclic elongation in uniaxial and biaxial directions. More importantly, the potential of this structure for on-skin applications was further demonstrated through epidermal biofuel cell fabrications, in which the bioenergy from sweat metabolites can be collected.

**Figure 14:**
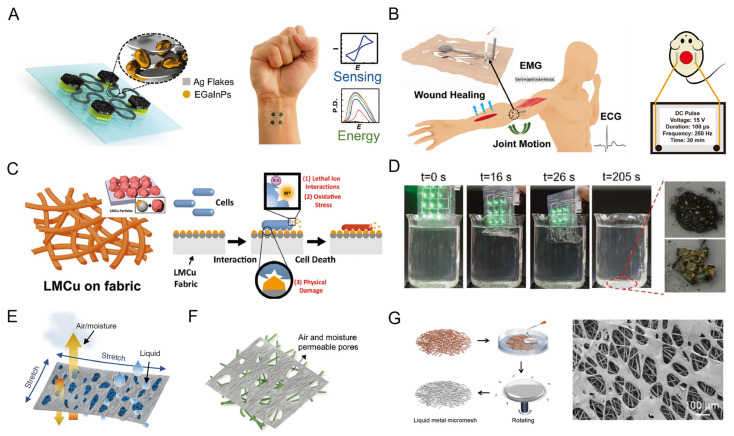
The LM-based electronics for other biomedical and sustainable applications. (**A**) Schematic of the LM island–bridge structure for wearable electrochemical devices (**left**), and the photograph of this device for on-skin sensing and energy applications [117]. Copyright: (2020) WILEY-VCH. (**B**) Schematic illustration of LM-based adhesive composite in the applications of wound healing, ECG, and joint motion monitoring (**left**), and the wound healing experiments on the back of mice with pulse electrical stimulation (**right**) [118]. Copyright: (2020) WILEY-VCH. (**C**) Schematic of LM–copper coating on a fabric (**left**, inset: the LMCu patterns on the fabric) and the proposed antimicrobial mechanism of LMCu coated on the fabric [119]. Copyright: (2021) WILEY-VCH. (**D**) Photographs of the recycling process for LM-based LED array [121]. Copyright (2019) WILEY-VCH. (**E**) Schematic illustration of the LM fiber mat via pre-stretching processing [57]. Copyright: (2021) Springer Nature. (**F**) Schematic illustration of porous LM super-lyophilic scaffold via pre-stretching [122]. Copyright: (2021) WILEY-VCH. (**G**) The fabrication of intrinsic highly permeable LM micromesh [123]. Copyright: (2022) American Chemical Society.

In addition, the wound healing and anti-microbial applications for epidermal protection are significant, especially in the post-pandemic era (COVID-19). An LM-based wet-adhesive electronics that can conformally adhere to the skin during a 48-hour wearing period with sports and showers (Figure 14B) was constructed in [118]. The biocompatible and self-adhesive property of LM-based electrodes enables the acceleration of wound healing through stable pulsed electrical stimulation. Compared with untreated areas, the wound area with electrical stimulation was much smaller and showed a more mature epidermis after 10 days. This further proves the promising potential of LM-based electronics in epidermal medical healthcare. In addition, for on-skin antimicrobial and antiviral applications, Gallium–LM particles were applied to reduce Cu ions for the formation of LMCu particles, which exhibit exceptional adhesion to fabrics and antibacterial, antiviral, and antifungal properties (Figure 14C) [119]. The LMCu coatings presented a thickness of several micros and were proved to contain three crystalline substances, i.e., Cu_2_O, Cu, and CuGa_2_. Coated with LMCu, the fabrics displayed broad-spectrum efficacy against Gram-positive and Gram-negative bacterium, fungi, and respiratory RNA human viruses.

In addition, the increasing electronic waste has gained considerable attention and urgent requirements for recyclable, degradable electronics [120]. LM possesses the intrinsic characteristic of recyclability through mechanical, chemical, or electrochemical treatment in the aforementioned oxidation layer removal process. By employing a degradable elastomer that serves as a structure supportive component for LM-based materials, the whole device can exhibit easy access to recyclable electronics. A water-soluble PVA/fructose substrate was applied to LM-based LED arrays (Figure 14D) in [121]. After 205 s of immersion in water, the whole film was completely dissolved and the LED chips, LM droplets, and PVA solution could be recollected separately.

The gas permeability of LM-based epidermal electronics is of significance for on-skin moisture comfort for long-term applications. Although LM is an intrinsically impenetrable barrier for gases (air and water vapor), porous LM conductive networks have been developed with textile or scaffold structures. An LM fiber mat was fabricated to achieve high permeability and stretchability via a pre-stretching process to a strain of 1800% (Figure 14E) in [57]. In this process, the original planar LM film was transferred to a mesh-like porous structure for gas and moisture permeability. A similar methodology was applied in an LM super-lyophilic fibrous scaffold with a high mass loading of LM by the wetting-enhancement of Ag (Figure 14F) in [122]. Through a simple pre-stretching, a porous structure of LM can be formed for permeability. To achieve the gas permeability without any mechanical stimulus, an LM nanomesh was introduced onto electrospun microfibers to realize intrinsic conductive and gas permeability, simultaneously, (Figure 14G) in [123]. By dropping LM on the SEBS scaffold and controlling the rotation speed of the composite, the permeability and conductivity of LM nanomesh can be coordinated.

## 5. Conclusions and Perspectives

To summarize, LM, especially for Ga-based alloys, with high conductivity and excellent stretchability provides a huge potential for next-generation epidermal healthcare devices. For stretchable electronics in the epidermal system, the ‘*BEER*’ requirement, i.e., biocompatibility, electrical elasticity, and robustness, is proposed in this review and for the guidance of future epidermal devices and systems. Before the discussion of the fabrication strategies of LM, the fundamental physical, chemical, and biocompatible properties of LM were thoroughly discussed. Consequently, four dominant LM strategies for the fabrication of LM electronics through size effect and surface reduction processes were comprehensively illustrated. Through these methodologies and technologies, a variety of on-skin healthcare applications of LM-based electronics was exhaustively discussed in five parts, i.e., electrodes for biomedical signal collections, interconnectors for functional circuits, mechanical sensors with various mechanisms, thermal management, and other biomedical sustainable applications. Although landmark progress has been achieved and widespread applications have been explored in epidermal healthcare, there are still several bottleneck challenges that limit the further development of LM-based electronics. Here, we list some major challenges and potential solutions as follows:

1. Leakage and encapsulation: Without the protection of an elastomer sheath or encapsulation, the leakage of LM would easily occur in deformations or under pressure. This has been a tricky issue in the practical application of LM-based electronics for a long time, especially for epidermal uses. Although the Ga-based LM was proved to be low cytotoxic, the residual LM after use and the leakage during dynamic deformations would affect the electrical resistance of the circuit or cause a short circuit. There are three main strategies for the leakage inhibition of LM, i.e., encapsulation, structure design, and size effect. The most common way to prevent the leakage of LM is based on physical encapsulation with elastomers. The encapsulation layers serve in the form of a condensed elastomer film [95], electrospun mat [124], and sheath for the core conductive fibers [78]. Second, other nanomaterials and novel structures have been introduced to LM conductive networks, such as the carbon nanofiber protection layer on LM particles in [125] and the microgrooves design for the abrasion resistance of LM in [126]. In addition, the size effect of LM was developed very recently, and the LM leakage issue can be addressed in an LM-elastomer mixture with the LM sized <5 μm [127]. The continuous rupture of LM particles upon mechanical stimuli can be prevented due to this size effect.
2. The mechanism of the modification of low viscosity LM: As discussed above, one of the effective methods for the surface tension reduction of LM is the mixture of LM and other rigid metal particles, including iron, copper, and nickel. The schematic model of the composites’ microstructure was provided to illustrate how the additive metal particles were wrapped with the gallium oxidation layer in [128]. In addition, the oxide skins of LM were continuously broken up through repetitive mechanical stirring to form Ga_x_O_y_ particles as well as internal air holes [129]. However, there is no sufficient evidence to illustrate the mechanism of viscosity regulation and the interactions between Ga_x_O_y_ components, Ga and In atoms through SEM, X-ray photoelectron spectroscopy (XPS), and energy-dispersive spectrometry (EDS). The potential solutions would go alongside other advanced characterization methodologies, including molecular dynamics simulation, atom probe tomography, etc.
3. The robust interfaces between LM-based circuits and semiconductor chips: Interconnection plays a significant role in robust on-skin electronics, and the interconnection enhancement between an LM and commercial semiconductor chips could definitely promote the development of hybrid electronics that could be potential candidates for high-density, multifunctional, and smart on-skin electronics. The current strategies involve developing intrinsically sticky conductors by compositing LM particles and adhesive elastomer [105,127]. LM-rich and elastomer-rich areas were formed on the surface of this composite. In addition, a biphasic LM with a mixture of liquid and crystalline solids was applied for a reliable low-resistance interface with rigid electronics in [130]. Other strategies that can realize high-precision circuits without crosstalk or short-circuiting upon dynamic deformations should be further studied.
4. The package of the whole epidermal healthcare system: LM-based electronics present tremendous potential in preventative medicine in the post-pandemic period. Nevertheless, an epidermal system that fuses the function and information among all LM-based epidermal devices is needed. In the future, a package of LM-based healthcare systems could be achieved. When patients encounter some general sickness or are willing for a health status check, LM-based healthcare devices could collect their physiological signals by cloud-medicine-assisted wearable devices efficiently and immediately. In these cases, the cooperation of various sensors, electrodes, signal collection and processing, and wireless connections should be extensively considered.

## Data Availability

Not applicable.
